# Gold Nanocluster Decorated Polypeptide/DNA Complexes for NIR Light and Redox Dual-Responsive Gene Transfection

**DOI:** 10.3390/molecules21081103

**Published:** 2016-08-20

**Authors:** Qi Lei, Jing-Jing Hu, Lei Rong, Han Cheng, Yun-Xia Sun, Xian-Zheng Zhang

**Affiliations:** Key Laboratory of Biomedical Polymers of Ministry of Education & Department of Chemistry, Wuhan University, Wuhan 430072, China; rachel914rae@163.com (Q.L.); lunalu025@163.com (J.-J.H.); ronglei@outlook.com (L.R.); chm128256@163.com (H.C.)

**Keywords:** NIR-responsive, redox-responsive, nuclear localization signal, endo/lysosomal escape, gene delivery

## Abstract

Endo/lysosomal escape and subsequent nuclear translocation are recognized as the two major challenges for efficient gene transfection. Herein, nuclear localization signal (NLS) peptide sequences and oligomeric lysine sequences were crosslinked via disulfide bonds to obtain glutathione (GSH) reducible polypeptide (pNLS). The pNLS could condense DNA into compact positive-charged complexes with redox sensitivity, and then gold nanoclusters (AuNC) were further decorated to the surface via electrostatic interactions obtaining versatile pNLS/DNA/AuNC complexes. The AuNC could generate reactive oxygen species (ROS) under NIR-irradiation and accelerate the endo/lysosomal escape of the complexes, and then the pNLS sequence degraded by GSH in cytoplasm would release the DNA and facilitate the subsequent nuclear translocation for enhanced gene transfection.

## 1. Introduction

Gene therapy is considered as a promising treatment for many serious diseases, including cancer, and mainly restricted by the success of gene delivery [1,2]. During the delivery process, the efficient gene compaction, cross-membrane delivery and unpacking of cargoes in cytoplasm/nuclei for gene expression are believed as major obstacles [3,4]. In order to overcome these barriers, many efforts have been devoted to developing efficient and versatile gene vectors, resulting in promising outcomes [5,6]. Among them, cationic peptide was of great interest due to their inherent biocompatibility and variety of functionalization [7,8]. For example, cell penetration peptide (CPP) and nuclear localization sequence (NLS) were vastly used in non-viral gene carrier decoration [9,10]. Meanwhile, ascribed to their electropositive structure, CPPs and NLSs were also cross-linked by sensitive linkers to form intracellular environment-responsive polypeptides with enhanced gene delivery capacity [11]. Taking advantage of the reductive environments in tumor cells, Zhang et al. formed a series of reductive-sensitive polypeptides (RSPs) by cross-linking oligopeptides using disulfide bond [12,13,14]. The RSP holds the merits of robust gene compact capability, and could rapidly release the loaded genes in tumor specific environments with reduced systemic toxicity [15,16,17]. Despite the enhanced gene packing and releasing capability, RSP still suffers from poor endo/lysosomal escape, and will be confined to the endo/lysosome, and eventually be eliminated by exocytosis [18,19].

Recently, photo-induced endo/lysosomal disruption opens a new era in the design of gene carriers [20,21,22,23]. By incorporating photosensitizers (PSs) into traditional gene vectors, the obtained carrier could disrupt the membrane structure of endo/lysosome under mild light irradiation by generating reactive oxygen species (ROS) [24]. Moreover, the low amount of ROS generated would only accelerate endo/lysosomal escape without destroying the loaded gene or evoking cell death. Liu et al. reported a light-responsive gene carrier for concurrently endo/lysosomal escape and gene release based on aggregation-induced emission (AIE) PSs and ROS-labile linkers [25]. Kataoka and coworkers introduced a dendrimeric PS to the surface of polycation/DNA complexes via electrostatic interactions [26,27]. However, these organic PSs often suffer from complicated synthesis and modification processes, poor hydrophilicity, and short exciting wavelength, which may seriously limit their further applications.

**Scheme 1:**
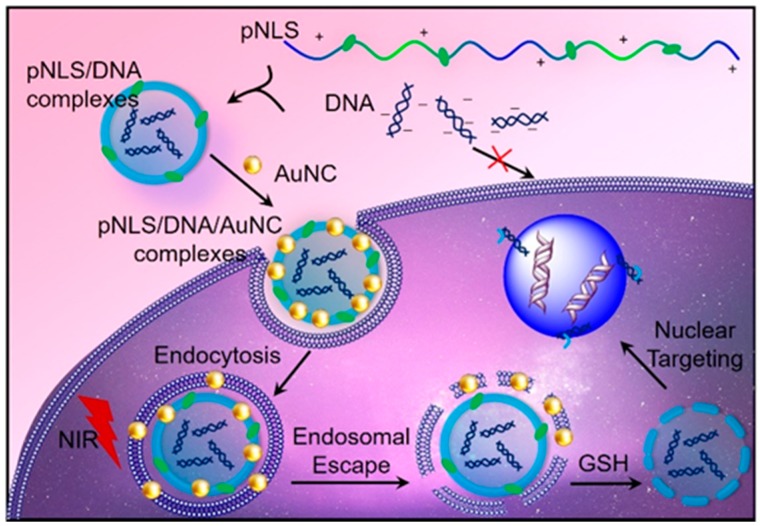
Facile construction of the NIR light-responsive and redox-sensitive pNLS/DNA/AuNC complexes for photo-triggered endo/lysosomal escape and gene delivery.

In this study, an inorganic PS, captopril-stabilized Au nanoclusters (Au_25_(Capt)_18_^−^, AuNC), was introduced into RSP for endo/lysosome escape enhancement, since the AuNC holds the merits of near-infrared excitation (808 nm) and satisfied hydrophilicity [28,29,30,31,32,33,34]. As shown in Scheme 1, electropositive peptide sequence (CKKKKKKC) and nuclear location sequence NLS (CPKKKRKVC) were cross-linked by disulfide bond to a obtain RSP (pNLS) with both gene compact and nucleus targeting capabilities. After loaded with desired gene, the pNLS was further decorated with AuNC to form the pNLS/DNA/AuNC complexes by electrostatic interactions. After having been internalized by cells via the endocytosis pathway, the AuNC could generate ROS and accelerate the endo/lysosomal escape of the pNLS/DNA/AuNC complexes under mild NIR-irradiation. Followed by the GSH induced disulfide bond breakage in cytoplasm, the complexes would release loaded genes and facilitate the subsequent nuclear translocation for enhanced gene transfection. Having circumvented the two major obstacles for gene delivery, endo/lysosomal escape and nuclear transportation, the pNLS/DNA/AuNC complexes exhibit remarkable promotion in gene transfection efficacy. Moreover, the convenient synthesis and decoration method also provide a new strategy for the construction of versatile gene delivery systems.

## 2. Results and Discussion

### 2.1. Synthesis of pNLS

An electropositive peptide sequence (CKKKKKKC) and nuclear location sequence (NLS) (CPKKKRKVC) were manually synthesized according to the Fmoc solid-phase peptide synthesis (SPPS) technique. The chemical structures of the peptides were confirmed by electrospray ionization mass spectrometry (ESI-MS), CKKKKKKC calculated 992.6, found 993.5 [M + H]^+^ and 497.4 [M + 2H]^2+^; and CPKKKRKVC calculated 1088.6, found 1089.6 [M + H]^+^ and 545.5 [M + 2H]^2+^ (Figure S1, Supplementary Materials). Then the two peptides were cross-linked in 30% dimethylsulphoxide (DMSO) at a feed mole ratio of 1:1. The molecular weight (Mw) of the obtained polypeptide (pNLS) was found to be 14,600 Da with the polydispersity index (Mw/Mn; PDI) of about 1.08 by size-exclusion chromatography and multiangle laser light scattering (SEC-MALLS), and the actual mole ratio of CKKKKKKC and CPKKKRKVC was 1.17:1 by ^1^H-NMR.

### 2.2. DNA Compaction Capability

**Figure 1:**
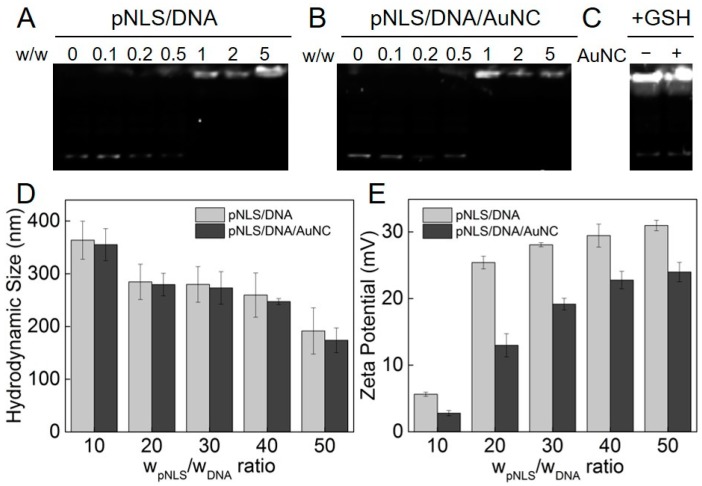
DNA compaction capability. Agarose gel electrophoresis retardation assay of (**A**) pNLS/DNA complexes and (**B**) pNLS/DNA/AuNC complexes in 10 mM phosphate buffered saline (PBS); and (**C**) complexes without or with AuNC at pNLS/DNA weight ratio of 10 in 10 mM GSH. Hydrodynamic size (**D**) and zeta potential (**E**) of pNLS/DNA complexes and pNLS/DNA/AuNC complexes at different weight ratios.

The capability to condense DNA into stable complexes is a prerequisite for cationic gene carriers. Once DNA binds with electropositive vectors, the obtained complexes should prevent DNA from enzymatic digestion and facilitate cell membrane entry. The DNA binding capacity of pNLS was investigated by agarose gel electrophoresis assay. As shown in Figure 1A, the migration of DNA was completely retarded at the *w*/*w* ratio of 1 (pNLS/DNA), owing to the robust DNA compaction capability of pNLS. This result also demonstrated that the electropositive pNLS could bind DNA effectively at very low weight ratios. Moreover, after the decoration of the AuNC, the complexes acquired demonstrated similar gene compact capability as pNLS (Figure 1B). This result indicated that the electronegative AuNC do not affect the DNA compact capability of pNLS.

Next, the degradation of the disulfide bond in pNLS was tested under simulated intracellular reductive environments, and GSH was employed as the reductive agent. Once the pNLS degraded into oligopeptides, the DNA binding capability of it would be seriously weakened. After being treated with GSH, bright bands of the dissociated DNA were found in pNLS/DNA and pNLS/DNA/AuNC complexes (Figure 1C). These results implied that the disulfide bonds in the two redox-responsive complexes could be efficiently degraded by the GSHin the reductive cytoplasm of tumor cells.

The small and uniform size is crucial for cell entry pathway of vector/DNA complex. Figure 1D displayed the hydrodynamic diameter of pNLS/DNA and pNLS/DNA/AuNC complexes at a *w*/*w* ratio (pNLS/DNA) ranging from 10 to 50 in 10 mM PBS. Declining trends in size were found for both complexes with the increased pNLS/DNA weight ratio. The decrease of the particle size was ascribed to the increased spare positive charge of the pNLS against the loaded DNA strings. At the same *w*/*w* ratio (pNLS/DNA), the particle sizes of pNLS/DNA/AuNC complexes are slightly smaller than that of pNLS/DNA complexes, which was attributed to the electrostatic effect between AuNC and the pNLS/DNA complexes, and the obtained complexes with diameter of 200–300 nm could be internalized through clathrin- or caveolae-mediated endocytosis pathways [35].

The positive charge of carrier/DNA complexes would assist their entry into cells owing to the electrostatic attraction against the negatively charged cell membranes. The zeta potential of pNLS/DNA and pNLS/DNA/AuNC complexes at different weight ratios were shown in Figure 1E. The zeta potential of the complexes vary from +5 to +30 mV. At the pNLS/DNA weight ratio of 10, the zeta potential of both complexes were about +5 mV, indicating the loose compaction between pNLS and DNA, which was consistent with the result of hydrodynamic size (larger than 300 nm), and with the increase of the *w*/*w* ratio, the zeta potential of the obtained complexes increased dramatically, and then reached a plateau at about +30 mV. The zeta potential of the pNLS/DNA/AuNC complexes was lower than that of the pNLS/DNA complexes, which was attributed to the decoration of the electronegative AuNC to the surface of the pNLS/DNA complexes. However, as demonstrated above, the electropositive property of pNLS/DNA/AuNC complexes still hold the merits of facilitating their cell internalization via electrostatic interaction-mediated uptake.

**Figure 2:**
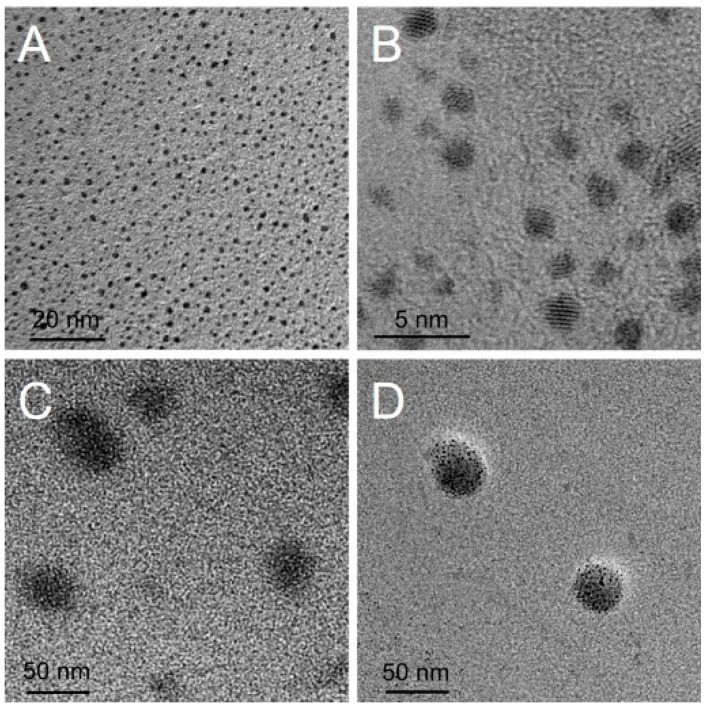
TEM images of (**A**,**B**) AuNC; (**C**) pNLS/DNA complexes at *w*/*w* ratio of 50:1; and (**D**) pNLS/DNA/AuNC complexes at *w*/*w*/*w* ratio of 50:1:5.

The size and shape of AuNC, the pNLS/DNA complexes, and the pNLS/DNA/AuNC complexes were evaluated by transmission electron microscopy (TEM). As presented in Figure 2A, the as-synthesized AuNC displayed homogeneous spherical particle shape with diameter of around 1 nm, and the high-resolution TEM image in Figure 2B showed the lattice image of AuNC, which indicated the successful synthesis of AuNC. Most of the pNLS/DNA complex particles in Figure 2C displayed uniform sphere morphology with diameter around 50 nm, which is smaller than the hydrodynamic size measured by dynamic light scattering (DLS). It is deduced that the TEM image was observed in a dry and vacuum state, while the hydrodynamic size obtained by DLS measurement was operated in a damp condition, resulting in a larger particle size. Meanwhile, as shown in Figure 2D, the pNLS/DNA/AuNC complexes exhibited satellite-like gold/complexes nanocomposites with a size around 50 nm, and the AuNC uniformly distributed on the surface of the complexes. The morphology of pNLS/DNA and pNLS/DNA/AuNC complexes indicates that the polypeptides could condense DNA compactly to form regular and tight spheres.

### 2.3. Cellular Uptake and Nuclear Translocation

**Figure 3:**
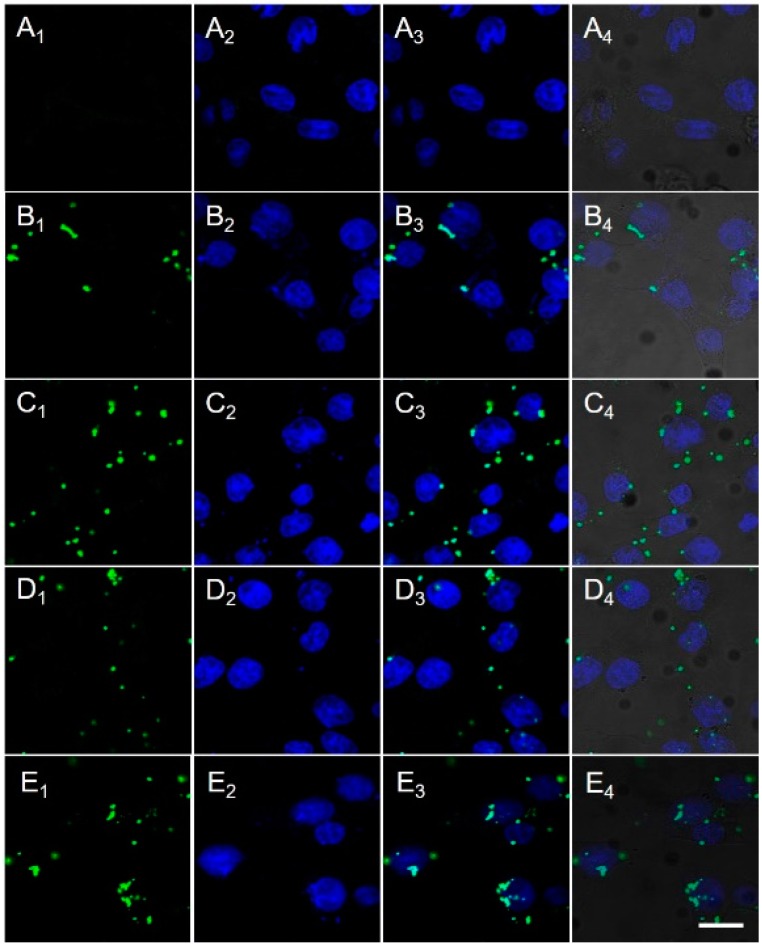
Cellular uptake of (**A**) naked DNA; (**B**) PLL/DNA complexes; (**C**) pNLS/DNA complexes; (**D**) pNLS/DNA/AuNC complexes; and (**E**) intracellular distribution of pNLS/DNA/AuNC complexes after NIR irradiation. The DNA stained with YOYO-1 are shown with green fluorescence in A_1_, B_1_, C_1_, D_1_, E_1_. The nuclei stained with Hoechst 33342 are shown with blue fluorescence in A_2_, B_2_, C_2_, D_2_, E_2_. The fluorescent channels are merged in A_3_, B_3_, C_3_, D_3_, E_3_. The fluorescent channels and bright field are merged in A_4_, B_4_, C_4_, D_4_, E_4_. Scale bar: 20 μm. (Green channel: excitation at 488 nm, emission at 500–550 nm; blue channel: excitation at 405 nm, emission at 440–460 nm).

In order to observe the intracellular fate of the delivered gene, DNA was labelled with YOYO-1, and the pNLS/DNA complexes at *w*/*w* ratio of 50:1 and the pNLS/DNA/AuNC complexes at *w*/*w*/*w* ratio of 50:1:5 were, respectively, prepared. PLL/DNA complexes at the *w*/*w* ratio of 50:1 were used as control. DNA labelled with YOYO-1 was displayed in Figure 3A_1_–E_1_, while the nucleus stained by Hoechst 33258 was shown in Figure 3A_2_–E_2_, and the merged fluorescent images were presented in Figure 3A_3_–E_3_, and the merged bright and fluorescent images were visible in Figure 3A_4_–E_4_. According to the confocal images in Figure 3, the cells treated with naked DNA displayed negligible green fluorescence, and the cells treated with different complexes showed obvious intracellular green fluorescence. Electronegative naked DNA was difficult to get into cells, but all complexes were able to transfer DNA into cells. The intracellular green fluorescence of pNLS/DNA complexes (Figure 3C) was stronger with that of PLL/DNA and pNLS/DNA/AuNC complexes (Figure 3B,D), attributing to their high zeta potential and strong electrostatic interaction with cell membrane. Meanwhile, the cellular uptake rate of the complexes was quantitatively analyzed by flow cytometry. Cells treated with naked DNA showed less than 7% of YOYO-1-positive cells and fairly low intracellular mean fluorescence intensity, but cells treated with all three complexes exhibited over 38% YOYO-1-positive cells (Figure S2, Supplementary Materials). In addition, the mean fluorescence intensity of YOYO-1 in cells of all three complexes measured by flow cytometry displayed similar tendency to what observed by CLSM. As demonstrated above, the pNLS/DNA complexes, pNLS/DNA/AuNC complexes and PLL/DNA complexes all hold great capability in cell entry via electrostatic interaction with cell membrane. However, after 4 h co-incubation, the green fluorescence of DNA in all complex-treated cells were mainly distributed in the cytoplasm and yet displayed no overlap with the blue fluorescence of the nuclei. These findings indicated that at current stage, the DNA loaded in all three complexes were entrapped in endo/lysosomal entrapment and result in limited nuclear translocation. However, when treated with NIR irradiation, cyan fluorescence (the overlapping of blue and green florescent signals) emerged in the nuclear region of the pNLS/DNA/AuNC complex-treated cells (Figure 3E), indicating the nuclei accumulation of the DNA. This finding manifested that, under NIR illumination, the pNLS/DNA/AuNC complexes can effectively mediate endo/lysosomal escape and nuclei import of DNA.

### 2.4. NIR-Responsive ROS Generation and Endo/Lysosomal Damage

**Figure 4:**
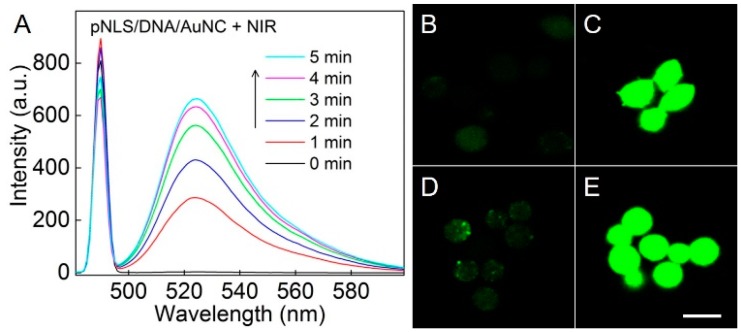
(**A**) ROS detection by DCFH in solution containing pNLS/DNA/AuNC complexes under NIR irradiation for different period. ROS detection by DCFH-DA in cells treated with (**B**) AuNC; (**C**) AuNC + NIR irradiation; (**D**) pNLS/DNA/AuNC complexes; and (**E**) pNLS/DNA/AuNC complexes + NIR irradiation. Scale bar: 20 μm. (Green channel: excitation at 488 nm, emission at 500–550 nm).

To verify the endo/lysosomal escape mechanism of the pNLS/DNA/AuNC complexes, ROS generation and ROS-induced endo/lysosomal damage under NIR irradiation were investigated, respectively. It has been reported that Au_25_(SR)_18_ clusters can produce ^1^O_2_ under NIR irradiation. Nonfluorescent dichlorodihydrofluorescein (DCFH) is a sensitive ROS indicator, which can be rapidly oxidized to fluorescent dichlorofluorescein (DCF) by ^1^O_2_. As shown in Figure 4A, without NIR irradiation, the pNLS/DNA/AuNC complexes solution demonstrated no fluorescence with the present of DCFH. However, after NIR irradiation, the fluorescence intensity of the solution was dramatically enhanced, indicating the generation of ROS. Same results were also found when DCFH co-incubated with AuNC under the presentation of NIR irradiation. Moreover, the fluorescence recovery process was completely inhibited by the addition of Vitamin C (a well-known ROS scavenger) (Figure S3, Supplementary Materials). These results demonstrated the robust ROS generation capability of AuNC and the pNLS/DNA/AuNc complexes under NIR irradiation. Subsequently, the intracellular ROS generation was also visualized by dichlorodihydrofluorescein diacetate (DCFH-DA), a cell-permeable ROS indicator. Without NIR irradiation, the intracellular green fluorescence in cells treated the AuNC and pNLS/DNA/AuNC complexes were weak and negligible, but strong green fluorescence was observed after NIR exposure (Figure 4B). This dramatic fluorescence change confirmed the intracellular ROS generation capability of AuNC and the pNLS/DNA/AuNC complexes.

**Figure 5:**
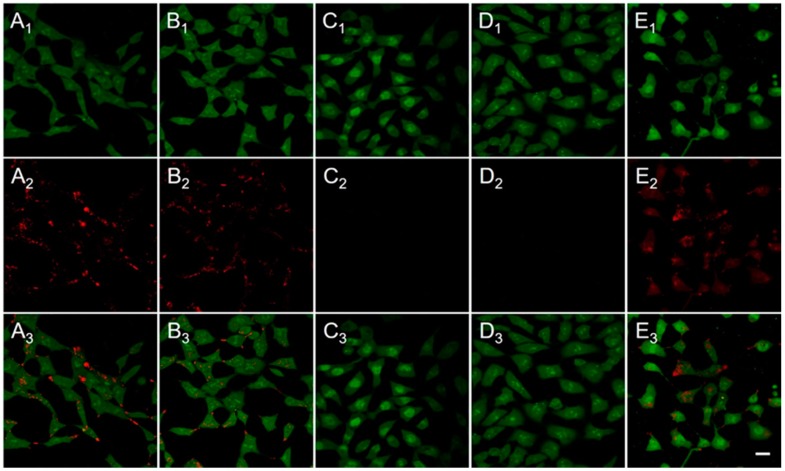
AO staining of cells treated with (**A**) AuNC; (**B**) pNLS/DNA/AuNC complexes; (**C**) AuNC + NIR irradiation; (**D**) pNLS/DNA/AuNC complexes + NIR irradiation and (**E**) pNLS/DNA/AuNC complexes + NIR irradiation + Vitamin C. The green fluorescent field for cytoplasm and nuclei (A_1_, B_1_, C_1_, D_1_ and E_1_), red fluorescent field for acidic organelles (A_2_, B_2_, C_2_, D_2_ and E_2_), and merged field of green and red fluorescent fields (A_3_, B_3_, C_3_, D_3_ and E_3_). Scale bar: 20 μm. (Excitation at 488 nm; green channel: emission at 500–550 nm; red channel emission at 630–670 nm).

Next, the ROS-induced damage of endo/lysosome was confirmed utilizing acridine orange (AO) as an indicator. AO displays green fluorescence in cytoplasm and nuclei, and exhibits red fluorescence in acidic organelles. Without NIR irradiation, cells treated with AuNC and pNLS/DNA/AuNC complexes displayed red and yellowish fluorescence in the edge of cells (Figure 5A,B), indicating the formation of endo/lysosomes in these cells and the endo/lysosomal entrapment of AuNC and pNLS/DNA/AuNC complexes. However, the intensity of red and yellowish fluorescence was remarkably weakened when the AuNC and pNLS/DNA/AuNC treated cells were exposed to NIR irradiation (Figure 5C,D), implying the dissociation endo/lysosomes by the ROS generated. Moreover, when incubated with ROS scavenger Vitamin C, the cells treated with s and NIR irradiation displayed red and yellowish fluorescence in cell edges (Figure 5E), which confirmed the endo/lysosome disruption capability of AuNC and pNLS/DNA/AuNC complexes by ROS generation under NIR irradiation. All these results manifested that the pNLS/DNA/AuNC complexes could generate ROS within cells as AuNC under NIR irradiation, and facilitate the endo/lysosomal escape during gene transportation process.

### 2.5. Cytotoxicity

**Figure 6:**
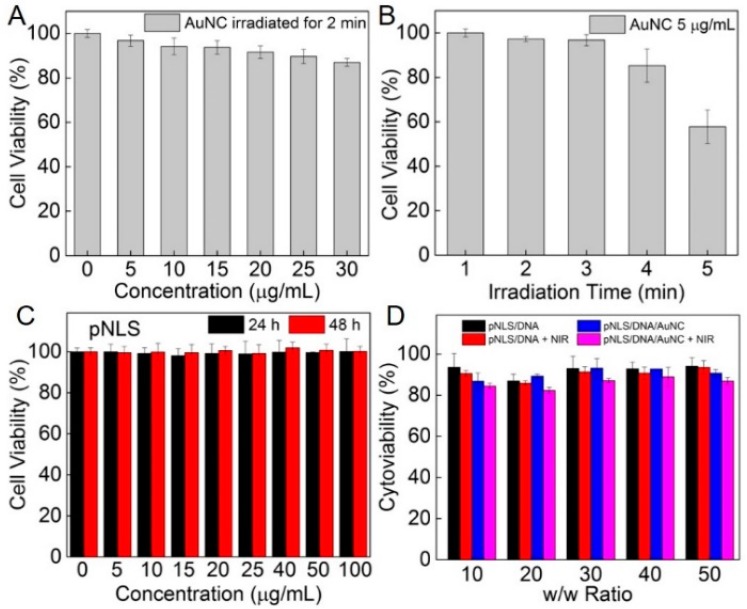
Cytotoxicity of AuNC under NIR irradiation (**A**) with different concentration and (**B**) for different exposure time; (**C**) cytotoxicity of cationic polypeptide pNLS; and (**D**) photo/dark toxicity of pNLS/DNA binary complexes and pNLS/DNA/AuNC complexes. Data are shown as mean ± S.D. (*n* = 6).

However, ROS is toxic to cells at a certain concentration. Therefore, it is important to control the generation of ROS that can only induce the disruption of endo/lysosomes without evoking cell death. AuNC exhibited negligible toxicity to the cells in dark, indicating its inherent biocompatibility as an inorganic nanoparticle (Figure S4, Supplementary Materials). After being irradiated with NIR light, AuNC showed concentration-dependent cytotoxicity (Figure 6A), but even at 30 μg/mL, the cell viability was still higher than 80%. Subsequently, the concentration of AuNC was fixed at 5 μg/mL, and the cells were irradiated for different time periods (808 nm, 1 W·cm^−2^). AuNC displayed obvious time-dependent cell inhibition (Figure 6B), and the cell viability decreased to about 60% after 5 min illumination, owing to the high concentration of ROS. Therefore, in the following assays, AuNC was fixed at 5 μg/mL and NIR irradiation was fixed at 1 W·cm^−2^ for 2 min to generate moderate ROS that can balance endo/lysosomal disruption and cell proliferation.

Meanwhile, the biocompatibility of pNLS was also investigated. As shown in Figure 6C, even at 100 μg/mL, pNLS showed no toxicity to the cells at both 24 h and 48 h. These results were attributed to the incorporation of reductive disulfide bonds in the polypeptides which can be cut off in reductive environments and allows a rapid degradation of pNLS into small non-toxic fragments, and the photo/dark cytotoxicity of the pNLS/DNA complexes and pNLS/DNA/AuNC complexes at different weight ratios was also low or negligible (Figure 6D). Therefore, both of them can be recognized as biocompatible gene delivery platforms (higher than 80% cell viability at various weight ratios).

### 2.6. Gene Transfection

**Figure 7:**
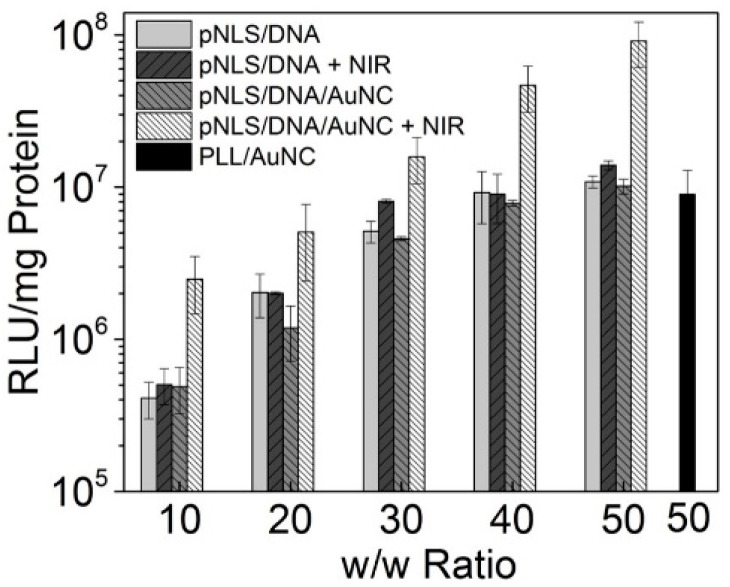
Luciferase expression in HeLa cells mediated by pNLS/DNA and pNLS/DNA/AuNC with or without NIR irradiation. PLL/DNA was used as control. Data are shown as mean ± S.D. (*n* = 3).

High-performance gene transfection is the foremost feature of the gene delivery carriers. To assess the transfection efficiency of different complexes, luciferase plasmid DNA pGL-3 was applied as model reporter genes. As shown in Figure 7, without NIR irradiation, the transfection level of pNLS/DNA complexes and pNLS/DNA/AuNC complexes were similar at all *w*/*w* ratios tested, owing to their related capability in cell entry and endo/lysosomal escape. Without irradiation, the complexes may be trapped in the acid organelles, and ended in exocytosis pathway, then the gene transfection process was inhibited. However, after exposed to NIR irradiation, the transfection efficiency of pNLS/DNA/AuNC complexes were enhanced sharply, while pNLS/DNA demonstrated negligible changes. These findings indicated that, the decoration of AuNC to the surface of the pNLS/DNA complexes could result in enhanced gene delivery efficiency. Thus, by decorating the ROS generator AuNC to the surface of the pNLS/DNA complexes, the obtained pNLS/DNA/AuNC complexes could generate ROS to disrupt the acidic endo/lysosomes under NIR irradiation, mediate enhanced endo/lysosomal escape, then respond to the reducible cytoplasm, direct nuclear translocation for efficient gene transfection.

## 3. Materials and Methods

### 3.1. Materials

NucleoBond Xtra Maxi EF plasmid purification was bought from Macherey-Nagel (Düren, Germany). GelRed was obtained from Biotium (Hayward, CA, USA). Dulbecco’s Modified Eagle Medium (DMEM), fetal bovine serum (FBS), phosphate buffered saline (PBS), and 3-(4,5-dmethylthiazol-2-yl)-2,5-diphenyltetrazolium bromide (MTT) were provided by Invitrogen Corp (Carlsbad, CA, USA). A Micro BCA protein assay kit was obtained from Pierce. Molecular probes (Hoechst 33258, YOYO-1 iodide, DCFH-DA) were purchased from Beyotime Biotechnology (Haimen, China).

Dimethylformamide (DMF), dimethylsulphoxide (DMSO), diisopropylethylamine (DIEA), trifluoroacetic acid (TFA), piperdine, tetraoctylammonium bromide (TOAB), HAuCl_4_∙3H_2_O, captopril, sodium borohydride (NaBH_4_), phenol, and ethanedithiiol (EDT) were provided by Shanghai Reagent Chemical Co. (Shanghai, China) and used directly. *N*-Fluorenyl-9-methoxycarbonyl (Fmoc)-protected l-amino acids: Fmoc-Pro-OH, Fmoc-Lys(Boc)-OH, Fmoc-Arg(Pbf)-OH, Fmoc-Val-OH, Fmoc-Cys(Trt)-OH, and 2-chlorotrityl chloride resin (100–200 mesh, loading: 1.0 mmol∙g^−1^), o-benzotriazole-*N*,*N*,*N*′,*N*′-tetra-methyluronium hexafluorophosphate (HBTU), and thioanisole were obtained from GL Biochem Ltd. (Shanghai, China) and used as received. Hyperbranched 25 kDa polyethyleneimine (PEI 25k) and polylysine (PLL, 15k) were purchased from Sigma-Aldrich (Shanghai, China) and used as received. Other reagents were of analytical grade and used as received.

### 3.2. Synthesis of Peptide Sequences

The peptide sequences (CPKKKRKVC and CKKKKKKC) were manually synthesized according to standard solid-phase methodologies based on Fmoc chemistry. Briefly, in the presence of DIEA/HBTU, amino acids were conjugated to a 2-chlorotrityl chloride resin (1.0 mmol·g^−1^) step-by-step. 20% piperidine/DMF (*v*/*v*) solution was added to remove the Fmoc groups in the sequence. The ninhydrin assay was applied to monitor the coupling efficacy of each step. After the removal of the last Fmoc groups in N-terminal, the peptide was entirely deprotected and cleaved from resin by using a 10 mL cocktail of TFA/phenol/thioanisole/EDT/deionized water (85:7.5:2.5:2.5:2.5 *v*/*v*/*v*/*v*/*v*) for 90 min at room temperature. The collected solution was concentrated by evaporation in vacuum and then dropped into cold diethyl ether and stored at −20 °C. The precipitate was centrifuged and dried under vacuum. And the product was dissolved in distilled water and then lyophilized and stored at −20 °C. The purity of the peptide was determined by high-performance liquid chromatography (HPLC, Prominence LC−20A, Shimadzu, Kyoto, Japan) with a C18 reversed-phase column by using a linear gradient from 95% to 5% of H_2_O/acetonitrile containing 0.1% trifluoroacetic acid at 1 mL·min^−1^ for 30 min. The purity of the peptides was at least 90%. The molecular weights of the peptides were measured by electrospray ionization mass spectrometry (ESI-MS, LCQ Advantage, Finigan, Santa Clara Valley, CA, USA).

### 3.3. Synthesis and Characterizations of Disulfide Linked pNLS

According to the literature, the reduction-sensitive pNLS was obtained by oxidative polycondensation from CPKKKRKVC and CKKKKKKC at feed mole ratio of 1:1 in PBS containing 30% DMSO (*v*/*v*) at room temperature for 96 h. The polypeptide was purified using dialysis membrane filters (MWCO: 3500 Da) to remove the uncrosslinked peptides and DMSO. The obtained pNLS was dissolved in distilled water and dialyzed for 24 h and then lyophilized before use for analysis and assay. Size-exclusion chromatography and multiangle laser light scattering (SEC-MALLS) analysis was applied to measure the molecular weights distribution of pNLS. A dual detector system consisting of a MALLS device (DAWNEOS Wyatt Technology, Santa Barbara, CA, USA) and an interferometric refractometer (Optilab DSP Wyatt Technology, Santa Barbara, CA, USA) was used HAc-NaAc buffer solution (0.03 M, pH 3.3) was used as eluent at a flow rate of 0.6 mL·min^−1^. The MALLS detector was operated at a laser wavelength of 690.0 nm.

### 3.4. Synthesis of AuNC

78.7 mg of HAuCl_4_∙3H_2_O and 126.8 mg of TOAB were dissolved in 10 mL of methanol. After 20 min of vigorous stirring, 5 mL of methanol containing captopril (1 mmol, 217.2 mg) rapidly poured into the solution. After further stirring for another 30 min, 5 mL of ice-cold deionized water containing NaBH_4_ (2 mmol, 75.6 mg) was rapidly injected in the mixture with vigorous stirring, which was continued for another 8 h. After that, the sample was centrifuged to remove the excessive Au(I) polymer. The supernatant was concentrated by rotary evaporation and then precipitated by ethanol. The precipitate was recrystallized for three times and dried in vacuum. The UV-VIS-NIR absorbance spectra of the AuNC (Figure S5, Supplementary Materials) were determinated by Lambda 35 UV/VIS spectrometer (Perkin Elmer, Waltham, MA, USA).

### 3.5. Cell Culture and Amplification of Plasmid DNA

Human cervix adenocarcinoma (HeLa) cells were incubated in DMEM supplemented with 10% FBS and 1% antibiotics (penicillin-streptomycin, 10,000 U·mL^−1^) at 37 °C in a humidified atmosphere containing 5% CO_2_. The luciferase reporter gene (pGL-3) was transformed in *Escherichia coli* JM109. First, the plasmids were amplified in LB media at 37 °C overnight at 250 rpm, then collected and purified by NucleoBond Xtra Maxi EF plasmid purification. Finally, the purified plasmids were dissolved in TE buffer solution at a final concentration of 200 μg·mL^−1^ and stored at −20 °C. The quality of each plasmid DNA (pDNA) was tested by agarose gel electrophoresis and ultraviolet (UV) absorbance at 260 and 280 nm.

### 3.6. Preparation of pNLS/DNA Complexes

The pNLS/DNA complexes at various weight ratios (*w*/*w*) ranging from 10 to 50 were prepared by adding dropwise 1 μg of pGL-3 DNA (200 μg·mL^−1^ in TE buffer solution) into an appropriate volume of pNLS solution (1 mg·mL^−1^ in 10 mM PBS), and then the complexes were diluted to a total volume of 100 μL with PBS and vortexed for 5 s. The mixtures were incubated at 37 °C for 30 min to form the complexes. All of the complexes were used immediately after preparation. The addition of AuNC decorations was in a similar way. AuNC with proper amount (5 μg) was added to the obtained pNLS/DNA complexes, and vortexed for 5 s, then incubated at 37 °C for 15 min to form the pNLS/DNA/AuNC complexes.

### 3.7. Agarose Gel Retardation Assay

The pNLS/DNA complexes at weight ratio ranging from 0.1 to 5 were prepared as mentioned above by adding dropwise 0.1 μg of pGL-3 DNA into an appropriate volumes of pNLS solution. The complexes were then diluted to a constant volume of 8 μL with PBS, and incubated at 37 °C for 30 min, and pNLS/DNA/AuNC complexes were prepared by adding a proper amount of AuNC (at DNA/AuNC weight ratio of 1:5). Subsequently, the samples were loaded onto the 0.7% (*w*/*v*) agarose gel containing GelRed and with Tris-acetate (TAE) running buffer at 80 V for 60 min. DNA was visualized under a UV lamp in the Vilber Lourmat imaging system (Paris, France).

To evaluate the ability of DNA release from pNLS/DNA complexes in vitro, reduced glutathione (GSH) was used for simulating the reductive environment of cytoplasm. The pNLS/DNA complexes and pNLS/DNA/AuNC complexes at a weight ratio of 10:1 and 10:1:5 were subsequently incubated with equivalent 10 mM GSH at 37 °C for 5 min. Then the samples were electrophoresed on the 0.7% (*w*/*v*) agarose gel containing GelRed and with Tris-acetate (TAE) running buffer at 80 V for 60 min. After that, DNA was visualized under a UV lamp in the Vilber Lourmat imaging system (Paris, France).

### 3.8. Particle Size and Zeta Potential Measurements.

The particle size and zeta potential of the complexes were measured with a Nano-ZS ZEN3600 (Malvern Instruments Ltd., Malvern, UK) Instruments at 37 °C. The pNLS/DNA binary complexes and pNLS/DNA/AuNC complexes were prepared as aforementioned. Next, the complexes were diluted to 1 mL volume with deionized water for particle size and zeta potential measurements.

### 3.9. Morphology

The morphologies of free AuNC in deionized water, pNLS/DNA complexes at *w*/*w* ratio of 50:1 and pNLS/DNA/AuNC complexes at *w*/*w*/*w* ratio of 50:1:5 in 10 mM PBS were observed by transmission electron microscopy (TEM, JEM-2100, JEOL Ltd., Tokyo, Japan). The complexes were prepared as described above. The TEM samples were prepared by dropping the obtained complexes solutions onto a copper mesh, and then drying under the infrared light before measurement.

### 3.10. Cellular Uptake Assays

The complexes transferred to cytoplasm and nucleus was observed in HeLa cells by the confocal laser scanning microscope. The DNA was labelled with YOYO-1, then the naked DNA, PLL/DNA, pNLS/DNA, pNLS/DNA/AuNC were co-incubation with HeLa cells for 4 h. The medium was removed and the cells were washed three times with PBS, followed nucleus staining with Hoechst 33342 for 15 min. The fluorescence was visualized on a confocal laser scanning microscope (CLSM, C1-Si, Nikon, Tokyo, Japan) (excitation wavelength: 408 and 488 nm, emission band pass: 430–470 and 500–550 nm), and the quantitative data were analyzed on a flow cytometer (BD FACSAria C6, Franklin Lakes, NJ, USA). For pNLS/DNA/AuNC complexes and NIR irradiation group, the cells were pre-incubated with pNLS/DNA/AuNC complexes for 4 h, washed with PBS, and irradiated with NIR laser (808 nm, 1 W·cm^−2^, 2 min) in fresh complete medium. Then the cells were further incubated in 37 °C for another 1 h before nuclear staining and CLSM observation.

### 3.11. ROS Detection

The ROS generation property of pNLS/DNA/AuNC complexes was identified using the ROS sensor DCFH-DA by means of fluorescence spectrum as well as CLSM. For the fluorescence spectrum, DCFH-DA was first converted to DCFH by NaOH treatment and then mixed with sample solutions (pNLS/DNA/AuNC at *w*/*w*/*w* ratio of 50:1:5, and AuNC was fixed at 10 μg·mL^−1^, DCFH was fixed at 0.5 μM). The mixed solutions were exposed to 808 nm NIR light irradiation for different time intervals at the power density of 1 W·cm^−2^. The fluorescence changes in the solutions were recorded at the preset time with an excitation wavelength of 488 nm and emission wavelength at 530 nm. PBS and AuNC with 100 μM Vitamin C (VC) were used as negative control. For CLSM observation, HeLa cells were incubated with AuNC and pNLS/DNA/AuNC for 4 h. Then, the medium was replaced and the cells were washed three times. DCFH-DA containing DMEM (10 μM) was added and further incubated for 30 min. Light irradiation was performed subsequently (808 nm, 1 W·cm^−2^, 2 min). The cells were observed via CLSM as soon as possible (excitation wavelength: 488 nm, emission band pass: 500–550 nm).

### 3.12. Disruption of Endo/Lysosome

HeLa cells were incubated with AuNC and pNLS/DNA/AuNC for 4 h. Then the medium was replaced and the cells were washed three times. After the light irradiation (808 nm, 1 W·cm^−2^, 2 min), the cells were stained with acridine orange (AO, 5 μM) for 10 min and washed with PBS for three times. Finally, the cells were observed via CLSM as soon as possible (excitation wavelength: 488 nm, emission band pass: 500–550 nm and 630–670 nm).

### 3.13. Cytotoxicity Assays

The cytotoxicity of AuNC without NIR irradiation and pNLS was estimated in HeLa cells by the MTT assay, respectively. In brief, HeLa cells were seeded in a 96-well plate at a density of 6000 cells per well and incubated in 100 μL DMEM containing 10% FBS for 24 h. Then, AuNC and cationic polypeptide pNLS at different concentrations were added to the wells. After 24 h or 48 h, 20 μL MTT (5 mg·mL^−1^ in PBS buffer solution) was added to each well and further incubated for 4 h. After that, the medium was replaced by 150 μL DMSO. The absorbance of the DMSO solution in the wells at the wavelength of 490 nm was measured by a microplate reader (Model 550, Bio-Rad, Berkeley, CA, USA) to determine cell viability. The relative cell viability was calculated as (OD_570sample_/OD_570control_) × 100%, where OD_570control_ was obtained in the absence of vectors and OD_570sample_ was obtained after co-incubation with materials.

The photo/dark toxicity of pNLS/DNA complexes and pNLS/DNA/AuNC complexes were also evaluated. After incubating the cells with 200 μL complete medium containing complexes for 4 h, the medium were replace by fresh medium and the cells of irradiation groups were illuminated with NIR laser (808 nm, 1 W·cm^−2^, 2 min). The cells were further incubated for 24 h. The amount of DNA was fixed at 0.2 μg per well. Then the cytotoxicity of the complexes was measured using the same protocol as mentioned above. The phototoxicity of AuNC was also measured in a similar way.

### 3.14. Transfection Assays

The in vitro transfection efficiency of complexes was evaluated in HeLa cells. The pNLS/DNA complexes were prepared at various weight ratios (pNLS/DNA) ranging from 10 to 50, and for pNLS/DNA/AuNC complexes, the weight ratio of DNA/AuNC was fixed at 1:5 (*w*/*w*). PLL/DNA at *w*/*w* ratio of 50 was applied as a control. The cells were seeded in the 24-well plate at a density of 6 × 10^4^ cells per well and cultured with 1 mL DMEM containing 10% FBS at 37 °C until reaching about 80% confluence. Then the cells were cultured with complexes in DMEM containing 10% FBS for 4 h at 37 °C. After that, the medium was replaced with fresh complete medium, and the cells in irradiation groups were treated with NIR laser (808 nm, 1 W·cm^−2^, 2 min). Then all of the cells were further cultured for 24 h. For luciferase assay, the medium was discarded and cells were washed with PBS, then the cells lysates were collected by treating with 200 μL reporter lysis buffer (Pierce). The relative lightunits (RLUs) were measured with a chemiluminometer (Lumat LB9507, EG and G Berthold, Bad Wildbad, Germany). The total cellular protein was measured by the BCA protein assay kit (Pierce) and luciferase activity was defined as RLU per mg protein.

## 4. Conclusions

In this work, a versatile gene delivery platform was constructed for NIR-triggered endo/lysosomal escape, GSH-triggered gene release, and NLS-mediated nuclear translocation. By using the ROS generation capability of AuNC, we obtained a promising carrier/gene/PS complexes that can generate ROS to disrupt endo/lysosomes under NIR irradiation. Interestingly, by modulating the NIR irradiation intensity and period, the obtained pNLS/DNA/AuNC complexes would only destroy the acidic organelles without evoking proliferation inhibition of the irradiated cells. Unlike PS decorated on previous photo-controlled gene delivery systems with drawbacks of poor hydrophilicity and short exciting wavelength, inorganic PS AuNC holds the merits of good hydrophilicity and NIR excitation capability. The long excitation wavelength at 808 nm with deep tissue penetration made AuNC a potential NIR light-responsive modifier for clinical trials. Meanwhile, disulfide linker with reduction-responsive breakage resulted in rapid gene release in the cytoplasm and the NLS sequence directed the liberated gene to the nuclei for successful gene transfection. This gene delivery platform that uses AuNC as the ROS generator may provide a new idea for photo-induced gene delivery with previous manners of spatiotemporal control.

## Supplementary Materials

The following are available online at http://www.mdpi.com/1420-3049/21/8/1103/s1. Chemical structure and mass spectra of peptide sequences, flow cytometry profiles, ROS detection by DCFH in solution, dark toxicity of AuNC and UV-VIS-NIR absorption of AuNC.
