# Supplementary material for: Gold Nanocluster Decorated Polypeptide/DNA Complexes for NIR Light and Redox Dual-Responsive Gene Transfection
Source: Molecules. 2016 Aug 20;21(8):1103. doi: 10.3390/molecules21081103 (PMC6273015; doi:10.3390/molecules21081103)
Supplement: Supplementary file 1 [file molecules-21-01103-s001.pdf]

Supplementary Materials: Gold Nanocluster Decorated Polypeptide/DNA Complexes for NIR Light and Redox Dual-Responsive Gene Transfection

Qi Lei, Jing-Jing Hu, Lei Rong, Han Cheng, Yun-Xia Sun and Xian-Zheng Zhang 1. Heading

Main text paragraph. Citing a journal paper [1]. And now citing a book reference [2]. Main text paragraph. Main text paragraph.

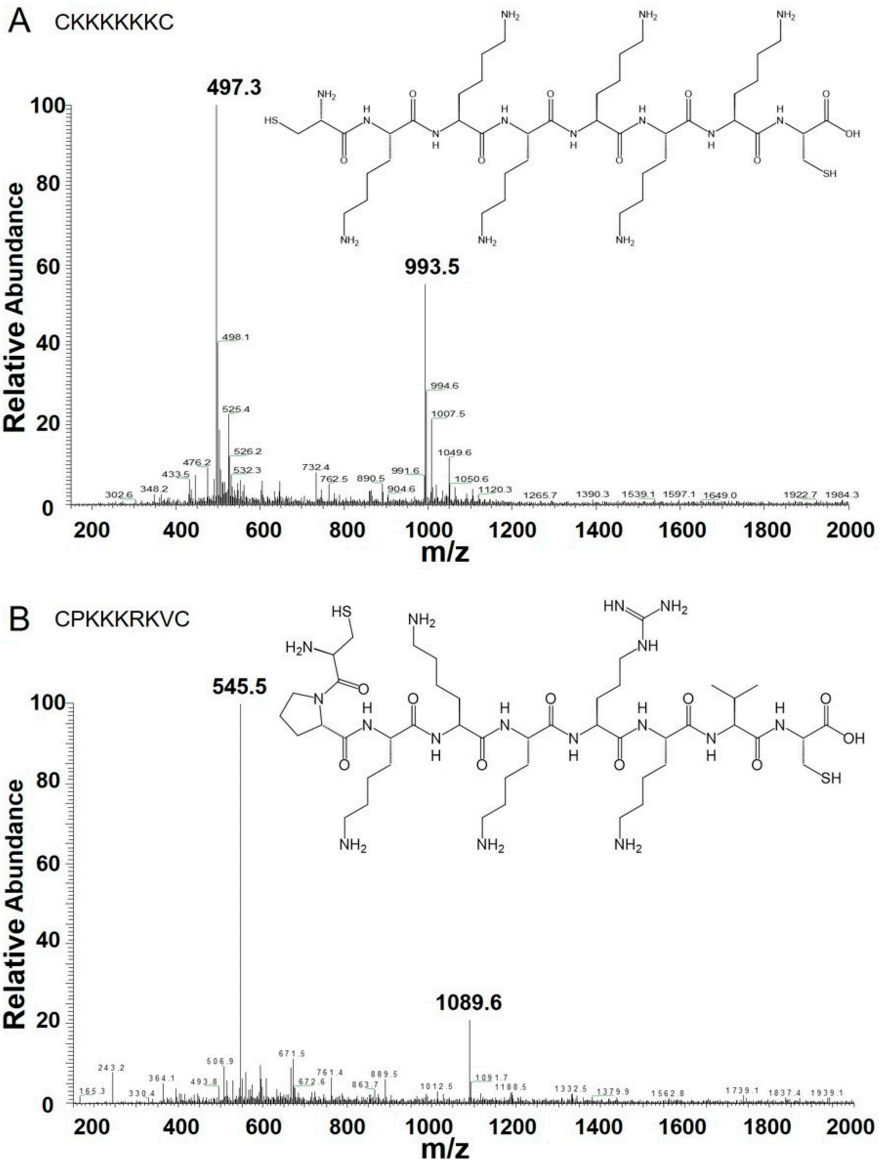

Figure S1. Chemical structure and mass spectra of (a) CKKKKKKC and (b) CPKKKRKVC peptide sequences.

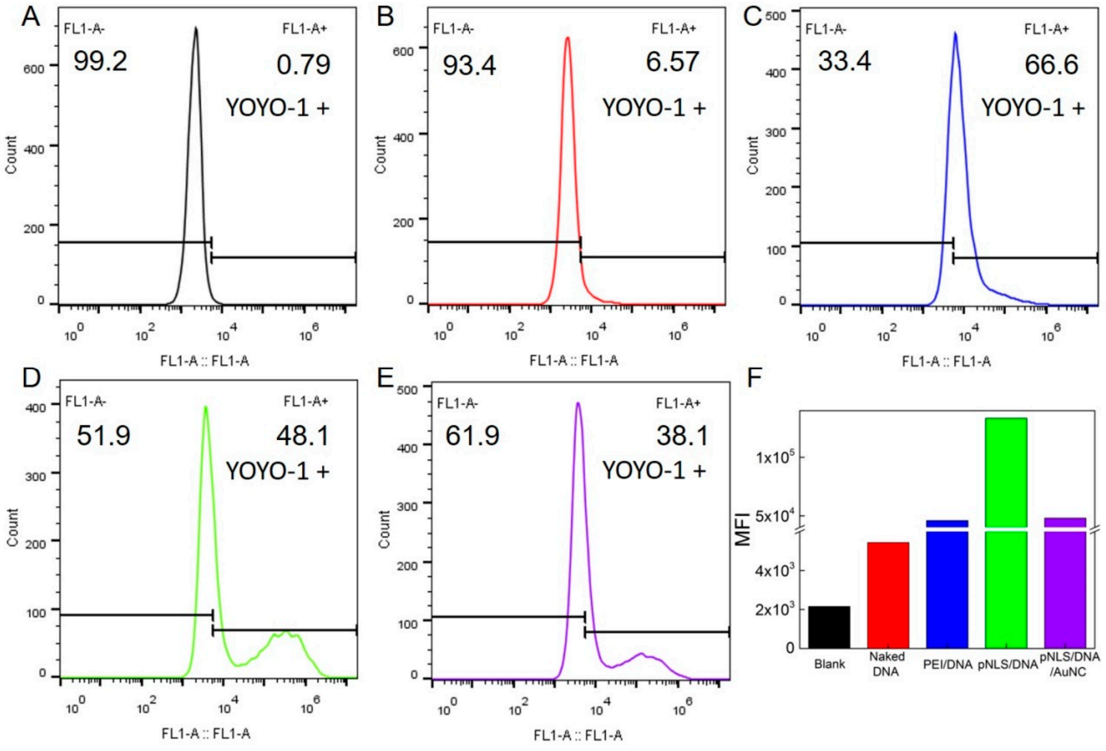

Figure S2. Flow cytometry profiles of (A) blank cells, cell treated with (B) naked DNA, (C) PLL/DNA, (D) pNLS/DNA binary complexes and (E) pNLS/DNA/AuNC ternary complexes at 4 h. (F) The mean fluorescence intensity of YOYO-1 in cells after different treatment.

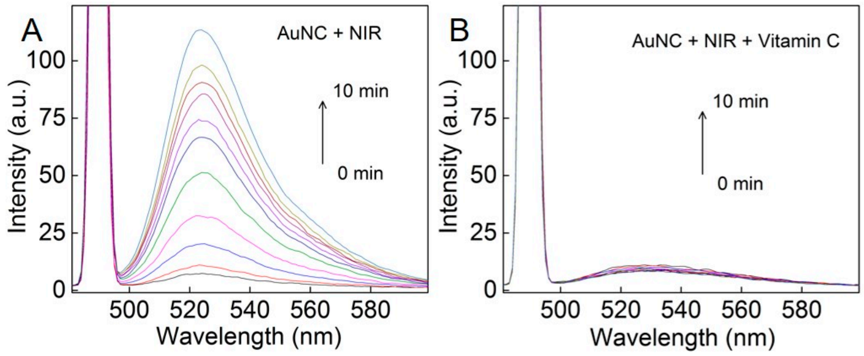

Figure S3. ROS detection by DCFH in solution containing (A) AuNC and (B) AuNC + Vitamin C under NIR irradiation for different time.

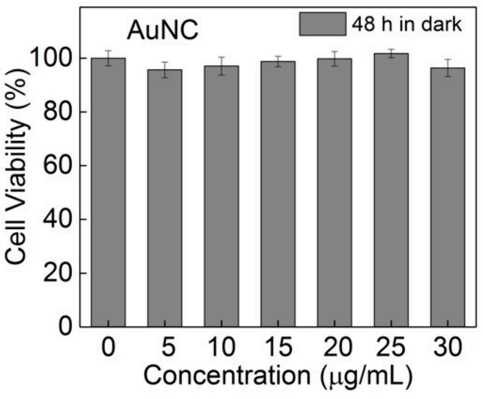

Figure S4. Dark toxicity of AuNC at different concentration for 48 h. Data are shown as mean ± S.D. (n=6).

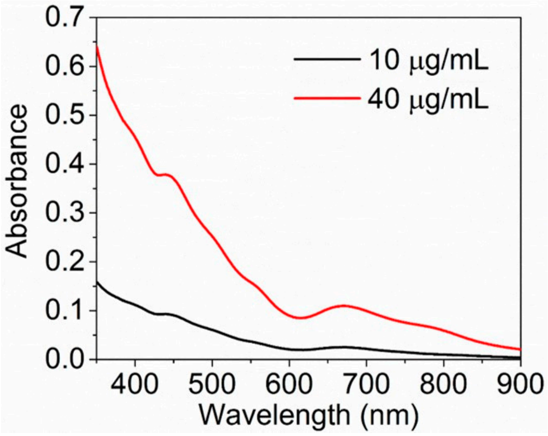

Figure S5. UV-VIS-NIR absorption of AuNC at the concentration of 10 μg/mL and 40 μg/mL
